# Advances in Probiotic and Prebiotic Applications for Sustainable Aquaculture: Harnessing Their Roles in China’s Marine Pasture Restoration and Health

**DOI:** 10.1155/anu/5011739

**Published:** 2026-08-21

**Authors:** Lishuko Ng’onga, Kwaku Amoah, Xiaopiao Zhong, Yong Zhong, Vicent Michael Shija, Peter Mrope, Yu Huang, Bei Wang, Xiao Jin, Jia Cai

**Affiliations:** ^1^ Shenzhen Institute of Guangdong Ocean University, Shenzhen 518120, China; ^2^ College of Fisheries, Guangdong Ocean University, Zhanjiang 524088, China, gdou.edu.cn; ^3^ Guangdong Provincial Key Laboratory of Aquatic Animal Disease Control and Healthy Culture, Key Laboratory of Control for Diseases of Aquatic Economic Animals, Key Laboratory of Marine Ecology and Aquaculture Environment of Zhanjiang, Zhanjiang 524088, China; ^4^ Southern Marine Science and Engineering Guangdong Laboratory, Zhanjiang 524002, China, sysu.edu.cn; ^5^ Guangxi Key Laboratory of Aquatic Biotechnology and Modern Ecological Aquaculture, Guangxi Academy of Marine Sciences, Guangxi Academy of Sciences, Nanning 530022, China, gxas.cn

**Keywords:** *Bacillus* species, China marine ranching, ecosystem restoration, immunomodulation, marine pollution, sustainable aquaculture practices

## Abstract

Aquaculture is the fastest‐growing food‐producing sector globally, yet intensive farming in China has led to severe ecological degradation of coastal marine pastures, including habitat loss, disease outbreaks, and dysbiosis of cultured species. Probiotics (beneficial live microorganisms) and prebiotics (non‐digestible substrates that promote beneficial microbiota) offer eco‐friendly alternatives to antibiotics and chemicals, aligning with China’s “Blue Granary” and marine ecological restoration initiatives. Recent advances demonstrate that multi‐strain probiotics (including *Lactobacillus*, *Bacillus*, and marine‐derived *Vibrio* spp.) and prebiotics (including mannan oligosaccharides, fructooligosaccharides, and seaweed‐derived polysaccharides) significantly enhance growth performance, immune responses, and disease resistance in key Chinese aquaculture species such as sea cucumber (*Apostichopus japonicus*), shrimp (*Litopenaeus vannamei*), and large yellow croaker (*Larimichthys crocea*). These biotics modulate gut and water microbiota, improve nutrient utilization, suppress pathogenic *Vibrio* populations, and mitigate environmental stress. In marine ranching and pasture restoration projects, probiotic‐prebiotic synbiotics have accelerated the recovery of degraded seaweed beds and benthic communities while reducing harmful algal blooms (HABs) and organic sediment loads. This review synthesizes evidence from Chinese and international studies (2018–2025) and highlights successful field‐scale applications in Bohai Bay, the Yellow Sea, and the South China Sea restoration zones. Integrating probiotics and prebiotics into feed and water management supports antibiotic‐free aquaculture, enhances carbon sequestration in marine pastures, and advances China’s goals of ecological civilization and sustainable blue economy.

## 1. Introduction

China’s large population and status as a major maritime power are complemented by the vital role of marine fisheries in ensuring food security. A national‐level perspective for analyzing, comprehending, and strategically planning ocean development is, therefore, indispensable for bolstering the marine economy and fortifying maritime influence [1]. The global importance of marine ecosystems and the profound impact of human activities upon them are undeniable [2]. Rapid population growth and densely populated coastal zones have intensified reliance on marine resources, which are defined here as marine systems where the ocean depth exceeds 50 m [3]. Marine pasture development holds great potential for conserving aquatic biological resources, improving marine water quality, and rehabilitating ecological environments. This innovative fishery production model is essential for optimizing the sector’s industrial structure and accelerating the transition from traditional methods. Concurrently, establishing national Marine Pasture Demonstration Zones can attract private investment, thereby promoting the rapid development of China’s marine pasture initiatives.

To address environmental degradation and resource constraints within the mariculture sector, China’s Ministry of Agriculture released the National Marine Pasture Demonstration Zone Construction Plan (2017–2025). This plan aims to drive sustainable growth by enhancing the marine ecosystem and promoting a shift towards more sustainable, integrated, and eco‐friendly marine fisheries practices. Subsequent meetings in 2021, which ratified the 14th 5‐year plan and the outline of the 2035 Vision Goals, underscored the urgent need to accelerate the growth of marine pastures and support the sustainable development of mariculture. The scientific and rational management of marine resources necessitates active human intervention within closed marine pastures. However, the marine environment faces increasing sustainability risks due to industrialization, tourism, maritime traffic, and global warming. Such anthropogenic and climatic changes can disrupt critical symbiotic relationships, ultimately affecting the health, physiology, behavior, and ecology of marine organisms [4].

Considering the oceans’ paramount significance to humanity and the mounting pressures they face, it is appropriate to determine and prioritize ocean health, particularly those broadly categorized as “marine pollution” (MP). MP adversely impacts both the environment and human health, causing increasing perturbation of the maritime environment and its biota. Pollutants can induce numerous biological effects, including metabolic dysfunction, genetic damage, and phenological harm. Furthermore, urbanization and industrialization have led to severe groundwater exploitation. This has resulted in declining water tables, large‐scale depression cones, land subsidence, and seawater intrusion, posing severe challenges for future groundwater resources [5]. Epidemic events can be catastrophic for host populations and may pose a threat to the affected species, triggering imbalances in the marine ecosystem.

Antibiotics have long been used to enhance ocean health, but they are now increasingly criticized due to the significant health hazards they pose to both humans and aquatic animals. In view of this, antibiotic use is increasingly being replaced by alternatives such as probiotics and prebiotics. These have gained attention as effective methods for controlling bacterial, viral, and parasitic infections in marine fish and shellfish [6–8]. According to the definition of the Food and Agriculture Organization (FAO) and the World Health Organization (WHO), probiotics are “live microorganisms which, when administered in an adequate amount, exert a beneficial effect on the health of a consumer” [9]. Merrifield et al. [10] proposed a revised definition of probiotics in aquaculture, considering the distinct traits of aquatic ecosystems when contrasted with terrestrial environments; thus, probiotics were characterized as either live or dead particles of microbial cells administered through food or rearing water. By fostering a beneficial microbial balance, probiotics enhance the host’s physiological resilience, leading to improved disease resistance, growth performance, feed efficiency, and a more robust response. There are some specific microbes shown to aid in the breaking down of waste or pollutants to enhance water quality, a process referred to as bioaugmentation or bioremediation [11]. Contrarily, prebiotics are indigestible compounds that serve as food for intestinal flora [12]. For substances to meet the criteria for prebiotics, they must promote the growth of beneficial gut flora and resist breakdown or absorption in the upper gastrointestinal tract (GIT) [13]. As a result, modulating endogenous microbial communities through probiotic or prebiotic supplementation can significantly influence the immunoregulatory systems governing gut mucosal tolerance. This review aims to synthesize and analyze the potential benefits of introducing probiotics and prebiotics into marine pasture environments to enhance marine ecosystem health and productivity. Our review has identified a literature gap in evaluating disease management strategies within marine pastures and a shortage of studies on marine pasture policy. Furthermore, research on applying probiotics and prebiotics for direct marine ecological protection and establishing corresponding management systems remains limited. Therefore, this review discusses the relevance of probiotics and prebiotics in the context of China’s marine pasture development mode, construction status, and management measures. It also addresses the challenges associated with the existing development model and proposes potential solutions for future development.

## 2. Probiotics

### 2.1. Definition and Application

Microorganisms that confer beneficial effects on their host are known as probiotics, a concept that has gained prominence in recent years [14, 15]. El Saadony et al. [16] noted that probiotics are live microorganisms introduced into the GIT through food or water, which help restore the microbial balance and enhance overall health. The gut microbiota produces metabolites that regulate immune response and metabolism. Accordingly, modulating the intestinal microbiota has emerged as a novel and increasingly significant approach to enhancing health and immunity, attracting considerable scientific attention. For instance, the intestinal microbiota metabolizes short‐chain fatty acids (SCFAs). These SCFAs strengthen the epithelial barrier and can reach distant organs, act on antigen‐presenting cells, and help attenuate inflammation in various diseases [17].

### 2.2. Sources of Probiotics

Potential probiotic microbes can be isolated from various sources. These include the intestines of healthy fish, aquaculture water, sediment from culture tanks, other aquatic organisms, and various fermented food items [18]. While the effectiveness of commercial probiotics remains ambiguous in the existing scientific literature, research indicates that host‐derived probiotics have significant potential to modulate the microbiota of aquatic organisms ex vivo [19]. These commensal bacteria are integral to the innate immune defenses of healthy hosts [20] and offer a more targeted approach [21]. Non‐marine sources of probiotics include fruits, vegetables, cereals, legumes, and pulses. *Lactobacillus plantarum*, *Bifidobacterium animalis subsp lactis* (commonly referred to as *Bifidobacterium lactis*), and *Saccharomyces cerevisiae* are effective non‐dairy probiotic microorganisms. These probiotics can improve gut health, help manage inflammatory bowel disease (IBD), and support immune function. The effectiveness, however, depends on the viability and stability of the microorganisms within various food matrices, highlighting the need to maximize their health benefits [22]. In parallel, research has been conducted on probiotic microorganisms isolated from aquatic species, including shrimp and finfish, as well as from terrestrial animals and their cultures. Within aquaculture, the effectiveness of these probiotics in preventing and controlling infectious diseases has been evaluated. The use of such specialized sources can enhance growth performance, improve immune responses, and offer a sustainable alternative to antibiotics in aquaculture [19].

### 2.3. Selection of Probiotic Strains

**Figure 1:**
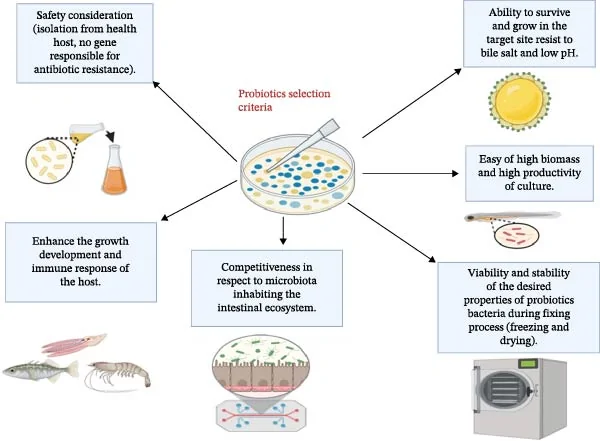
The selection criteria for probiotics.

The primary objective of probiotic application in marine species is indeed to conserve the beneficial ecological balance between commensal and pathogenic bacteria in their skin mucus and alimentary canals. By modulating the immune responses and improving disease resistance, probiotics can help maintain mucosal homeostasis [23]. This application should also safeguard the surrounding environment, such as water and sediment quality. Historically, the choice of probiotic bacteria has typically been made on an ad hoc, trial‐and‐error basis with a limited scientific rationale. This lack of stringent selection has led to many research failures. While established selection methods are available, they are not universally applicable due to the diverse host species and environmental contexts. Thus, the selection of potential probiotics and their mode of action must be clearly defined [24]. Key determinants of these selection criteria include biosafety considerations, production and processing methods, delivery systems, and the anticipated sites of microbial activity within the host. The selected probiotic strain ought to exert a verifiable beneficial effect on the host animal, such as increased growth or resistance to disease. Additionally, it is crucial that these strains remain effective during production, manufacturing, distribution, and storage [25]. Given that the beneficial effects of many species are species‐specific, it is ideal to isolate candidate bacteria from the same species for which they are intended to be used on. This approach guarantees a better biological fit for specific conditions in the animal’s digestive system [26]. When used as feed additives, probiotic cultures must withstand pelleting conditions, humidity during transportation and storage of feed, and potentially aggressive environments with the risk of exposure to mycotoxins or heavy metals. Probiotics added to feed and premixes should have a high viability period of at least 4 months. Formulations often require encapsulation to ensure an adequate amount of viable probiotic strains reaches the host’s GIT [27]. Safety, efficiency, and technical feasibility are vital to the selection process. Most importantly, selected probiotics should not harbor plasmid‐encoded antibiotic resistance genes. They should also tolerate a broad pH range and bile salt concentrations >2.5% [28]. Addressing these factors can enhance the effectiveness and reliability of probiotics in aquaculture and animal husbandry (Figure 1).

### 2.4. Types of Probiotics

Currently, the market offers a variety of commercial probiotic products derived from diverse sources, including bacterial strains, yeast, and microalgae. Among these, gram‐positive bacteria generally demonstrate greater efficacy than gram‐negative ones. The most widely recognized probiotic strains belong to the category of lactic acid bacteria (LAB) group, with notable examples including *Bifidobacterium* and *Streptococcus*. Although still a relatively novel concept in aquaculture, the use of probiotics has garnered significant interest for their potential to regulate physiological processes in aquatic species. Several strains, such as *Lactobacillus helveticus*, *Aeromonas media*, *Bacillus subtilis*, and *Enterococcus faecium*, have been identified as highly effective. Additionally, it is noteworthy that the GIT of fish and shellfish harbors gram‐negative facultative symbiotic anaerobes, including *Vibrio*, *Pseudomonas*, *Plesiomonas*, and *Aeromonas*, which may also serve as promising probiotic candidates [29].

#### 2.4.1. *Bifidobacterium* Species

Several species of the *Bifidobacterium* genus have been identified and approved as potential probiotics. These anaerobic bacteria, which produce antimicrobial proteinaceous substances, are part of the intestinal microbiota of fish and mammals [30]. For example, the probiotic *Bifidobacterium animalis* subsp. *lactis* BB‐12 (BB‐12) has been well established and is among the most studied due to its health benefits. This strain has been shown to enhance immune responses, assist in the prevention of diarrhea, and reduce the likelihood of constipation. Due to its widespread and clinical food industry use, understanding its developmental properties is crucial for any product based on this strain [31]. For efficient BB‐12 strain culture and preservation, the choice of culture media is essential. Among these compound reagents used in the preparation of the culture media, nitrogen plays a particularly significant role, serving not only as a growth precursor but also as a substrate for the bacterium’s metabolic pathways. This allows strain BB‐12 to produce diverse metabolites, such as vitamins, SCFAs, and antibiotic compounds, that may contribute to its health benefits [32]. Treatment with *Bifidobacterium lactis* BL‐99 significantly reduced intestinal inflammation in adult zebrafish (*Danio rerio*), increased the number of goblet cells, and produced several beneficial metabolites [33]. Immune cells have been reported to be activated by *B. longum* [34], and when supplemented at 10^7^ CFU/g in diets, *B. longum* improved *Penaeus vannamei*’s growth performance [35].

#### 2.4.2. *Enterococcus* Species

Enterococci, a group of LAB, are considered potential probiotic candidates for aquafeed. They exhibit resistance to stomach acidity and bile salts, possess strong epithelial binding properties, and show immunomodulatory and antagonistic effects against pathogens [36]. Their ability to produce bacteriocins makes them an attractive alternative to the classical antibiotics. For instance, *Enterococcus faecium* significantly decreased mortality in European eels (*Anguilla anguilla*) exposed to *Edwardsiella tarda* [37]. *Enterococcus casseliflavus* is a promising probiotic for rainbow trout culture due to its impact on immune stimulation and growth performance [38]. According to Akbari et al. [36], a bacteriocinogenic strain of *Enterococcus mundtii* was isolated from the intestinal contents of *Hemiodema spectabilis* collected from the Patagonian coast of Argentina. In this study, the marine‐derived strain demonstrated antibacterial activity, showing resistance to most antibiotics tested except cephalothin while exhibiting activity against various pathogens, including *Listeria innocua* and *vancomycin-resistant enterococci*. However, the ability of *Enterococcus* species to develop multiple antibiotic resistances has drawn increasing attention [39].

#### 2.4.3. *Lactococcus* Species

*Lactococcus lactis* is a major strain of LAB recognized as safe gram‐positive bacterium. It plays an important role in maintaining intestinal microbiota balance and promoting the immune function of animals [40]. *L. lactis* is well known for its special uses in improving animal feed, fermenting milk, and producing vaccines [41]. Supplementing *L*. *lactis* PH3‐05 at a dose of 10^6^ CFU/g diet significantly stimulated the growth, survival, and digestive status of *A. tropicus* larvae [42].

#### 2.4.4. *Lactobacillus* Species

*Lactobacillus* species are gram‐positive bacteria known to improve immune responses and guard against upper respiratory and gastrointestinal diseases. The protection of fish against pathogen infections is a key benefit of utilizing Lactobacillus [43]. Adhesion is regarded as a beneficial characteristic for a probiotic strain as it can improve the residency of probiotics in the gut and their interactions with the host’s epithelium and immune cells; Lactobacillus species are known to possess this particular trait [44]. Among them, *L. fermentum* strains have been reported to generate a variety of strong antimicrobial peptides usable as antibiotic substitutes or as food preservatives [45]. An investigation of *Lactibacillus plantarum* CLY‐05’s effects specifically on sea cucumber (*Apostichopus japonicus*) demonstrates increased body weight and improved non‐specific immune enzyme activities [46]. As a result of improving intestinal health and integrity, *Lactiplantibacillus plantarum* E2 significantly increased large yellow croaker (*Larimichthys crocea*) survival and growth rates. Its supplementation increased intestinal enzyme activities (amylase, trypsin, and lipase) and improved gut microbiota composition by decreasing harmful Sphingomonas while increasing beneficial *Lactobacillus* and *Pseudomonas*. In addition, E2 supplementation enhanced disease resistance against *Pseudomonas plecoglossicida*, suggesting its potential as a probiotic to promote the growth and health of large yellow croaker [47]. According to Feyereisen et al. [48], *Levilactobacillus brevis* is a heterofermentative, Gram‐positive LAB that thrives at pH 4–6 and 30°C. Additionally, it has been demonstrated that certain strains of *L. brevis* are able to produce biogenic amines, including tyramine and phenylethylamine [49]. The majority of *L. brevis* strains exhibit strong antibiotic and proteolytic activity as well as adhesive qualities to GIT cells [50]. Cell‐free supernatants of *L. brevis* isolates demonstrated strong immunostimulatory effects on Pikeperch and Carp immune cells [51]. Gram‐positive, nonspore‐forming, nonmotile, microaerophilic microbes with a high tolerance to acidic pH conditions make up the species *Lactobacillus delbrueckii* [52]. *L. delbrueckii* has an obligately homofermentative metabolism, producing primarily lactic acid, with an ideal growth temperature of 42°C [53]. Furthermore, strains of *L. delbrueckii* can be isolated from a variety of ecological sources, such as vegetables, dairy products, fermented foods, and human tracts [54]. According to Silvi et al. [55], *L. delbrueckii* shows a strong ability to colonize the gut of sea bass, altering the gut microbiota, which translates into higher chances of survival. *Cyprinus carpio* growth, gastrointestinal enzyme activities, and growth‐related gene expression can all be enhanced by supplementing *L. delbrueckii* in diets at ~1 × 10^6^ CFU/g [56].

#### 2.4.5. *Bacillus* Species

One of the most commonly used probiotic genera known to produce extracellular enzymes is the *Bacillus* species. These species are known to produce spores that are impervious to physical and chemical conditions; thus, they are able to withstand the harsh conditions met during feed processing. Juvenile coral trout grouper (*Plectropomus leopardus* E3S) have been shown to grow more when fed *Bacillus cereus* [57]. In a study conducted by Yao et al. [58], *Bacillus* strains were isolated from the intestinal tract of healthy large yellow croaker (*Larimichthys crocea*), and four strains with protease and lipase activities were identified. Among these, *Lysinibacillus* sp. strain LYD11 exhibited strong inhibitory activity against two major aquaculture pathogens, *Vibrio harveyi and Vibrio alginolyticus*. Additionally, strain LYD11 demonstrated effective coaggregation and adhesion exclusion capabilities, suggesting its potential as a promising probiotic candidate for disease prevention in aquaculture, particularly against common bacterial infections [58]. Also, *Bacillus licheniformis* ATCC 11946 supplementation at a dose of 10^8^ CFU/g diet was reported to enhance the growth, proximate body composition, serum and hepatic immune and antioxidant enzyme activities, intestinal morphology, and assembly of intestinal microbiota by yielding more beneficial bacteria and reducing pathogenic bacteria in *Litopenaeus vannamei*, which translated to shrimp protective capacity against *Vibrio parahaemolyticus* [59]. Numerous studies have identified the Gram‐positive bacterium *Bacillus subtilis* as a traditional probiotic for aquaculture species [60]. Research indicates that *B*. *subtilis* enhances host immunity against bacterial infections and viral diseases while also playing a role in immunomodulation [61]. *B*. *subtilis* strain HGCC‐1 has been shown to positively influence liver health in golden pompano by regulating the intestinal microbiota, reducing hepatic steatosis, and promoting growth performance [62]. It was found that dietary *Bacillus licheniformis* significantly improved the physiology and immunity of the sea cucumber (*Apostichopus japonicus*). Growth performance, digestive and immune enzyme activity, and *vibriosis* resistance were enhanced as a result of supplementation. By combining *Bacillus licheniformis* with herbal extracts, the microbial community was optimized by promoting beneficial bacteria and inhibiting pathogens, resulting in reduced cumulative mortality rates and improved overall health [63].

#### 2.4.6. *Clostridium* Species

*Clostridium butyricum* is primarily used in the treatment of intestinal inflammation associated with dysbiosis or imbalances in the intestinal flora [16]. The therapeutic benefits of *C*. *butyricum* include the repair of intestinal damage, enhancement of nutrient absorption and digestion, and modulation of the immune response [64]. Incorporation of *C. butyricum* at concentrations ranging from 10^7^ to 10^11^ CFU/kg improved the growth performance of giant yellow croakers (*Larimichthys crocea*) [65]. It was identified that *Clostridium species* were present in Pacific white shrimp (*Litopenaeus vannamei*), including *Clostridium subterminale*, *Clostridium beijerinckii*, *Clostridium butyricum* GC subgroup A, and *Clostridium bifermentans* GC subgroup A. *Clostridium* spp. were detected in 16 strains. In shrimp intestines, these anaerobic bacteria produce enzymes for carbohydrate and protein breakdown, which contribute to the absorption of nutrients [66].

#### 2.4.7. *Shewanella* Species

Numerous studies have highlighted the beneficial effects of *Shewanella* species as probiotics in aquaculture. For example, *S. putrefaciens* (strain Pdp11 or more recently as SpPdp11), isolated from healthy gilthead seabream, has shown probiotic potential for this species [67]. Additionally, *Shewanella* sp. MR‐7, isolated from the turbot fish gut, significantly enhanced their intestinal health [68]. In Pacific white shrimp, it improved growth performance, bolstered immunity, and increased the population of beneficial gut bacteria. Supplemented animals exhibited greater weight consistency and higher total protein concentrations, alongside elevated alkaline phosphatase activity and enhanced non‐specific immune responses. Furthermore, the administration of *S. putrefaciens* Pdp11 from the onset of exogenous feeding positively influenced the larval development of Senegalese sole (*Solea senegalensis*) [69]. Feeding *Shewanella haliotis* to *Litopenaeus vannamei* significantly increased respiratory burst activity [70] and *Shewanella colwelliana*, according to Jiang et al. [71], enhanced abalone’s innate immunity and tolerance to diseases.

#### 2.4.8. Yeast and Algal Species

In recent years, yeasts have gained increasing attention for their potential health benefits as probiotics. Beyond the well‐studied *Saccharomyces boulardii*, scientists are actively searching for new yeast strains with probiotic qualities, including species such as *Debaryomyces hansenii*, *Kluyveromyces marxianus*, *Yarrowia lipolytica*, *Pichia hudriavzevii*, and *Torulaspora delbrueckii*. These are isolated from diverse sources such as traditional fermented foods, the human gut, and natural environments. Research suggests that some of these yeasts could offer support in managing IBD, irritable bowel syndrome, skin conditions, and allergies. Yeast probiotics, such as *Debaryomyces hansenii*, are utilized in aquaculture to enhance growth, modulate the gut microbiota, and improve the health of farmed fish. They promote somatic growth, improve feed efficiency, and stimulate immune responses without disrupting the intestinal cell organization. *D*. *hansenii* has shown the ability to reduce opportunistic Proteobacteria while enhancing beneficial microbial populations, contributing to a healthier intestinal environment. This makes yeast probiotics a valuable addition to sustainable aquaculture feeding strategies [72]. Studies indicate that *S*. *cerevisiae* positively affects growth rates, feed efficiency, and water quality in various fish species. It works by stimulating the immune system, altering microbial metabolism, and competing with pathogens for resources [73]. Microalgae, such as *Dunaliella salina*, *Dunaliella tertiolecta*, *Isochrysis galbana*, and *Tetraselmis suecica*, have been shown to improve growth, health, and survival rates in aquatic animals. These algal probiotics contribute beneficial nutrients and enhance the immune response of cultured species. Their incorporation into aquaculture practices can improve the overall performance and suitability, underscoring their potential as effective probiotics alongside traditional bacterial strains [74].

### 2.5. Mode of Action of Probiotics

Probiotics play a crucial role in promoting fish health within aquaculture by engaging various biological pathways. They are vital for regulating the gut microbiota and maintaining microbial balance by facilitating the colonization of beneficial bacteria while inhibiting the growth of harmful pathogens through a process known as competitive exclusion. By obstructing pathogens’ access to essential nutrients and binding sites on the gut’s surface, probiotics significantly reduce the incidence of bacterial infections. In addition to combating infections, probiotics produce antimicrobial compounds, including bacteriocins and organic acids, that further suppress the growth of harmful bacteria. They also bolster the host’s immune system, enhancing innate and adaptive immune responses. The primary biological mechanisms by which probiotics exert their effects include increased adhesion to the intestinal mucosa, improved epithelial barrier function, inhibition of other pathogenic microbial adhesion, competitive exclusion of pathogenic microorganisms, production of antimicrobial substances, and modulation of the immune system [75].

The synthesis of inhibitory compounds is another critical aspect of probiotics’ functionality. Among various substances, probiotic bacteria generate bacteriocins, hydrogen peroxide, siderophores, lysozymes (LYZs), and proteases, all of which exhibit bactericidal or bacteriostatic effects on competing microbial populations [76]. Moreover, certain bacteria produce volatile fatty acids and organic acids, such as lactic, acetic, butyric, and propionic acids. These compounds can lower the pH of the gastrointestinal lumen, inhibiting the growth of opportunistic pathogenic microbes [77].

**Figure 2:**
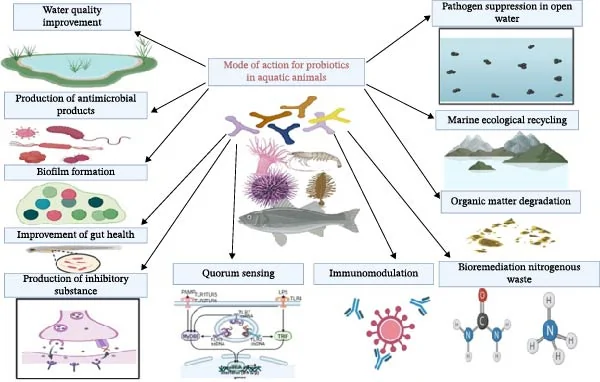
Mode of action of probiotics in aquatic animals.

Another critical mechanism of action is the competition for nutrients and available energy. The ability of microbial populations to compete with other organisms in the same environment for essential chemicals and energy sources is vital for their survival [6]. Various microorganisms, including the well‐known probiotic group of LAB, consume nutrients necessary for the growth of certain pathogens. This competitive dynamic can significantly influence the composition of the gut microbiota or the culture water of aquatic organisms. Improving water quality is another important aspect. Utilizing Gram‐positive bacteria, such as *Bacillus* species, can enhance the quality of aquatic systems. Unlike Gram‐negative bacteria, which tend to convert a larger proportion of organic matter into bacterial biomass or slime, *Bacillus* species are more efficient at converting organic matter into carbon dioxide [78]. Furthermore, some probiotic bacteria exhibit strong algicidal effects, particularly against various microalgal species. The application of nitrifying cultures in fish habitats also plays a crucial role in mitigating the toxicity of nitrite and ammonia [78]. Likewise, they bolster fish immune systems, aiding the development of natural resistance and improving the survival rates of fish larvae and post‐larvae. Ibrahem [79] highlights the varied stimulatory effects of probiotics on fish immune systems, including enhanced immune cell activity, antibody production, acid phosphatase (ACP) levels, LYZ activity, and the synthesis of antimicrobial peptides. Additionally, quorum sensing is a sophisticated communication mechanism employed by bacterial cells to interact both within their own species and with other species, particularly when their population density surpasses a specific threshold. This intricate process is initiated by the release of signaling molecules known as autoinducers [80]. These autoinducers bind to receptor proteins, triggering the quorum‐sensing transduction pathway. This pathway subsequently activates the expression of genes associated with pathogenicity, spoilage, and biofilm formation. It has been suggested that metabolites produced by probiotics may possess the ability to inhibit quorum sensing [81]. Through coordinated communication, bacteria can effectively regulate collective behaviors that enhance their survival and adaptability in various environments. This innovative approach underscores the potential of probiotics to improve fish health and contribute to sustainable aquaculture practices. Figure 2 illustrates the mode of action of probiotics in aquatic animals.

### 2.6. Beneficial Effects of Probiotics

#### 2.6.1. Growth Performance and Immune Response

Incorporating probiotics and prebiotics into aquaculture practices has been shown to increase growth rates and enhance feed conversion ratios (FCR). Probiotics facilitate better nutritional absorption and digestion, enabling farmed fish to utilize feed more efficiently and to accumulate greater biomass. Furthermore, probiotics contribute to the gut’s structural integrity by increasing the villus height and surface area, thereby enhancing nutrient absorption and digestion efficiency and improving growth performance. They also promote microbial balance and reduce intestinal inflammation, benefiting fish health and improving feed consumption [82] (Table 1).

**Table 1:** The beneficial effects of probiotics in fish.

Probiotics	Host animal species	Duration of experiment	Dosage	Region	Effects	References
*Bacillus subtilis* BS1 and *Lactobacillus plantarum*	Chinese Perch (*Siniperca chuatsi*)	8 weeks	1.0 × 10^8^ CFU/g	Huazhong, China	Enhance growth performance and gut health by regulating gut microbiota	[83]
*Saccharomyces cerevisiae* and *Lactobacillus bulgaricus*	Chelon ramada (*Mugil capito*)	60 days	*S. cerevisiae*, 4 g/kg diet + *L. bulgaricus*, 2 g/kg diet)	Kafr Elsheikh, Egypt	Improve growth, body composition, antioxidant defenses, and hepatic and intestinal health	[84]
*Bacillus subtilis*, *Bacillus licheniformis*, and *Trichoderma longibrachiatum*	Rohu (*Labeo rohita*)	8 weeks	0.25, 0.50, and 0.75 g/kg	Kasur, Pakistan	Enhance growth, immunity	[85]
*Bacillus halophilus*	Large yellow croaker (*Larimichthys crocea*)	60 days	10^8^ CFU/mL	Fujian, China	Improve growth, antioxidant immunity ability, and promote the expression of growth‐related metabolites	[86]
*Bacillus subtilis*, *Enterococcus* sp., *Lactobacillus casei*, *Lactobacillus rhamnosus*, *Saccharomyces cerevisiae*, and *Saccharomyces boulardii*	Pacific white shrimp (*Penaeus vannamei*)	75 days	5 × 10^9^ CFU/mL^−1^	Tamil Nadu, India	Enhance growth and immunity	[87]
*Lactobacillus plantarum* and *Saccharomyces cerevisiae*	Japanese grenadier (*Coilia nasus*)	120 days	1.0 × 10^8^ CFU/g *Lactobacillus plantarum: Saccharomyces cerevisiae*, 9:1	Yangzhong, China	Enhance lipid metabolism, suppress fatty acid synthesis, and reduce oxidative stress and inflammation	[88]
*Bacillus licheniformis*	Sea bass (*Lates calcarifer*)	8 weeks	1 × 10^6^/CFU/g^−1^	Santa Maria RS, Brazil	Improve growth, chemical composition of fish, activity of the liver, and digestive enzymes	[89]
*PrimaLac*	Caspian white fish (*Rutilus frisii kutum*)	45 days	1%	Tehran, Iran	Elevate immune markers for skin mucus	[90]
*Clostridium butyricum*	Large yellow croaker (*Larimichthys crocea*)	30 days	5 × 10^9^ CFU/g^−1^	Qingdao, China	Promote growth	[65]
*Clostridium butyricum*	Sea Cucumber (*Apostichopus japonicus*)	63 days	1%	Luoyang, China	Enhance growth promotion and nonspecific immunity	[91]
*Pseudoalteromonas piscicida*	Pacific white shrimp (*Litopenaeus vannamei*)	14 days	10 g/L	Dramaga Bogor, Indonesia	Improved growth performances, immune responses, and disease resistance	[92]
*Enterococcus faecium*	Shark catfish (*Pangasianodon hypophthalus*)	56 days	2.0 × 10^11^ CFU	Alexandria, Egypt	Modulates growth, digestive enzymes, immunity, hepatic antioxidant activity, and disease resistance	[93]
*Bacillus velezensis V4* and *Rhodotorula mucilaginosa*	Atlantic salmon (*Salmo salar* L.)	56 days	10^6^ CFU/g^−1^	Qingdao, China	Improved growth, immune response, antioxidant capability, and disease resistance	[94]
*Clostridium butyricum*	Giant river prawn (*Macrobrachium rosenbergii*)	60 days	2 × 10^9^ CFU/g^−1^	Khulna, Bangladesh	Promote growth performance, digestive enzyme activity, and immune response	[95]
*Bacillus megaterium PTB 1.4*	Catfish (*Clarias* sp.)	30 days	10^10^ CFU/mL^−1^	Bogor, West Java, Indonesia	Enhanced protease and amylase activities	[96]
*Debaryomyces hansenii CBS 8339*	Rainbow trout (*Oncorhynchus mykiss*)	37 days	10^6^ CFU/g^−1^	Plouzane, France	Promote larval development	[97]
*Bacillus licheniformis TC22*	Sea cucumbers (*Apostichopus japonicus*)	30 days	10^9^ CFU/g	Yantai, China	Improve immunity and disease resistance	[98]
*Bacillus subtilis* and *licheniformis*	Japanese eel (*Anguilla japonica*)	12 weeks	1 × 10^8^ CFU/g	Busan, Korea	Enhance growth	[99]
*Pseudomonas aeruginosa*, *Staphylococcus aureus*, *Salmonella typhi*, *Escherichia coli*, and *Candida albicans*	African catfish (*Clarias gariepinus*)	60 days	2 mL/kg	Addis Ababa, Ethiopia	Enhance microbial populations	[100]
*Bacillus* sp., *Streptococcus faecalis* and *Clostridium butyricum*	Nile tilapia (*Oreochromis niloticus*)	75 days	Basal diet + probiotics, at 10 g/kg	Mymensingh, Bangladesh	Improve gut microbiota and intestinal morphology	[101]

Probiotics also act as immunostimulatory agents, helping mitigate the effects of disease and reduce pathogen entry, thereby providing essential protection for cultured aquatic organisms [29]. Utilizing probiotics as immunostimulants is a highly effective strategy for enhancing the success of aquaculture operations. Research indicates that after being exposed to *Pseudomonas* and *Aeromonas hydrophila*, probiotic *Staphylococcus edaphicus* successfully boosts the immune response of Kelabau fish (*Osteochilus melanopleurus*), enhancing blood parameters and survival rates [102]. Furthermore, *Staphylococcus* sp. strain JC20 has demonstrated cellulolytic properties and probiotic potential in red tilapia without adverse effects [103] (Table 1).

#### 2.6.2. Probiotics on Water Quality Improvement

Ensuring high‐quality water is essential for the optimal performance of aquatic species, and probiotics are noted to play a crucial role in this process. To inhibit the growth of harmful bacteria, probiotics release various byproducts like lactic acid, hydrogen peroxide, and bacteriocins [104]. The hydrogen peroxide released by probiotic bacteria triggers chain reactions of superoxide anions, resulting in a powerful oxidizing effect with bactericidal properties [105]. Studies show that by maintaining pH and reducing harmful ammonia and nitrite levels, mixed *Bacillus amyloliquefaciens* probiotics significantly improve water quality during white shrimp aquaculture [106]. Adding *Bacillus subtilis* to shrimp farms at a concentration of 10^10^ CFU/mL has been shown to lower total ammonia levels and improve water quality [106]. A high concentration of probiotics in fish ponds plays a crucial role in balancing phytoplankton production and mitigating the accumulation of dissolved and particulate matter throughout the growing season [19]. Probiotics also release lactic acid, which can completely inhibit pathogens at 0.5% (w/v) and create an unfavorable environment for unwanted microbes in the culturing environment [107]. Also, probiotics can release nitric oxide (NO) that helps regulate biofilm formation [104]. In addition to these benefits, many probiotic bacteria have been found to have significant algicidal effects on microalgae [108]. Nitrifying bacteria play a crucial role in removing harmful ammonia and nitrite from water and improving the water’s overall microbial composition. In aquaculture water, aerobic denitrifiers are considered effective in converting nitrate and/or nitrite into N_2_ [109].

**Figure 3:**
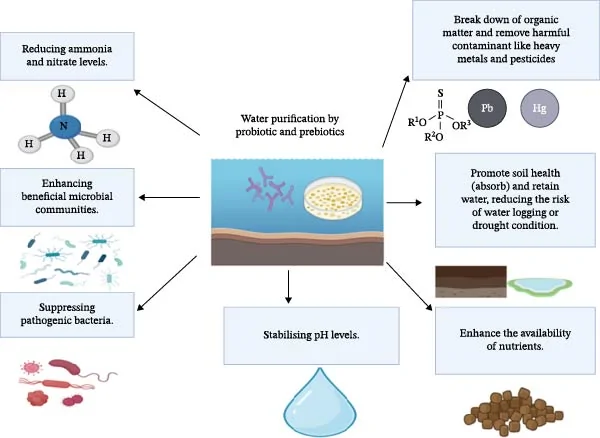
The positive impact of probiotics and prebiotics in water. This figure demonstrates the beneficial effects of incorporating probiotics and prebiotics into water sources.

Studies have shown that treating Penaeid culture with *Bacillus pumilus* can improve water quality measures and provide protection against marine pathogens, such as *Vibrio* spp. [110]. By producing spores and metabolites that inhibit pathogenic microbes, probiotics can effectively mitigate water contamination and maintain a suitable water quality for aquaculture activities. In conclusion, probiotic bacteria play a crucial role in enhancing aquaculture water quality by regulating algal growth and inhibiting the growth of pathogenic microbes (Table 1). This can lead to increased productivity and overall success in aquaculture operations [111]. The diagram presented in Figure 3 illustrates the positive impact of probiotics and prebiotics in water.

### 2.7. Probiotics and Bioremediation

**Figure 4:**
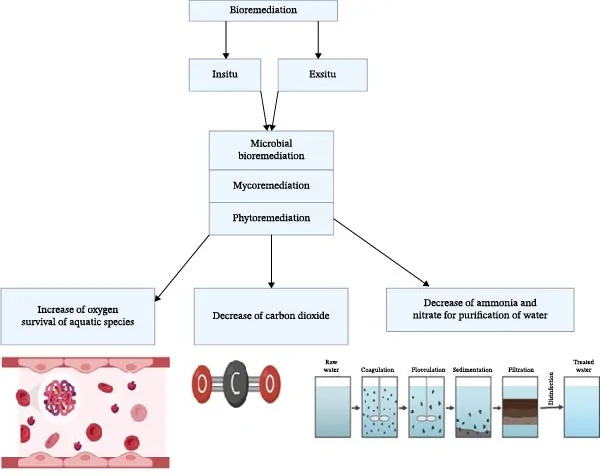
Illustration of the various methods of bioremediation, which use biological organisms to remove or neutralize contaminants in the environment.

Beyond serving as feed supplements, certain probiotics also function as bioremediation agents, breaking down toxic compounds in the environment. Bioremediation relies on microorganisms, including bacteria, fungi, yeast, and algae, as well as microbial byproducts, to degrade, reduce, or eradicate harmful environmental substances. In some cases, phytoremediation, which uses plants, is also employed in certain situations [112]. During bioremediation, microorganisms produce enzymes that transform toxic chemicals through biotransformation. This often leads to biodegradation, where pollutants are broken down into simpler structures, ultimately resulting in harmless and non‐toxic byproducts, a process referred to as mineralization. Several bacterial species, including *Bacillus*, *Pseudomonas*, *Acinetobacter*, *Cellulomonas*, *Rhodopseudomonas*, *Nitrosomonas*, and *Nitrobacter*, are recognized for their effectiveness in remediating organic waste [113]. For example, De Paiva‐Maia et al. [114] studied the effects of a commercial probiotic on bacterial and phytoplankton densities in intensive *Litopenaeus vannamei* farming using a recirculating method. In 2.6‐hectare ponds stocked at 98 shrimp/m^2^, probiotics were applied weekly starting 7 days before stocking and continued throughout the 16‐week experimental period. Probiotics were found to greatly influence the levels of heterotrophic bacteria and Pyrrophyta in sediment, ultimately enhancing the overall environmental conditions. In another study, Hassan et al. [115] examined two probiotic bacteria for bioremediation in earthen ponds stocked with *Pangasius sutchi*, *Catla catla*, and *Labeo rohita* for a year. Probiotic application reduced ammonia, nitrite, and phosphate levels in treated ponds, indicating effective remediation. The study showed that *Bacillus* spp. produced digestive enzymes such as protease, amylase, and lipase, which helped suppress pathogenic *Vibrio* species without harming shrimp post‐larvae. Figure 4 displays various types of bioremediation techniques.

### 2.8. Recommendation and Factors Affecting Probiotic Effectiveness

The type and quantity of probiotic strains used can significantly influence the effectiveness of probiotic formulations. Several factors contribute to the efficacy of these formulations, including the selection of optimal strains and the appropriate dosage that ensures enough viable cells. Formulations must be stored and handled according to the manufacturer’s instructions to preserve the viability of these live microorganisms as they are sensitive to adverse conditions such as temperature and light [116]. When administering probiotics, it is essential to avoid concurrent use of other medications and to ensure that the dilution water is free of disinfectants, like chlorine. It is recommended that animals receive the probiotic‐infused water within 6–12 h [26]. Furthermore, a 24–48 h waiting period is recommended after antibiotic or other antimicrobial treatment before initiating probiotic therapy. Generally, the most effective probiotic formulations contain a diverse array of ingredients, incorporating a greater number of microbial species. The method of administration is also crucial; common approaches include injection, ingestion, encapsulation, or immersion in water. In aquaculture, effective probiotics should exhibit strong adhesion or colonization capabilities or reflect the microbial communities in the fish mucosa [28, 117, 118].

It is important to recognize that probiotics designed for aquatic animals are distinct from those used in terrestrial systems due to fundamental ecological and physiological differences [56]. While certain bacteria may be harmful to one aquatic species, they can serve as beneficial probiotics for others. In recent decades, the performance of aquatic animals has seen significant improvement using this biological control agent. It is crucial to consider the optimal conditions for probiotics to survive, colonize, proliferate, and effectively provide their benefits to hosts in a specific environment. The notion of a ‘one size fits all’ approach does not apply to probiotics, as specific strains or species are required for target species in particular environments.

## 3. Prebiotics

### 3.1. Definition and Application of Prebiotics

Prebiotics are indigestible food components that confer health benefits to the host by promoting the growth and activity of beneficial bacteria in the colon, ultimately enhancing overall well‐being [6]. Many food oligosaccharides and polysaccharides are recognized for their prebiotic properties; however, “prebiotics” and “dietary fibers” are often used interchangeably, leading to confusion. It is important to note that not all carbohydrates in our diet qualify as prebiotics. The necessity of rigorously defining a food component as a prebiotic is underscored by researchers who emphasize that scientific evidence must demonstrate the component’s ability to withstand gastric acidity, enzymatic breakdown, intestinal digestion, and microbial fermentation [119]. Mechanistically, prebiotics function by serving as substrates for beneficial gut bacteria, specifically fostering their growth. The fermentation of prebiotics produces SCFAs, such as acetate, propionate, and butyrate, which offer a multitude of health benefits. These include lowering intestinal pH, providing energy to intestinal cells, and enhancing gut health by reducing susceptibility to infections. Furthermore, prebiotics improve nutrient digestion and absorption by increasing enzymatic activity and facilitating the uptake of essential minerals, including calcium, magnesium, and phosphorus. Prebiotics also play a crucial role in strengthening the intestinal barrier by modulating tight junction proteins, thereby reducing gut permeability and preventing the entry of pathogens and toxins into the bloodstream.

### 3.2. The Selection of Prebiotics

**Figure 5:**
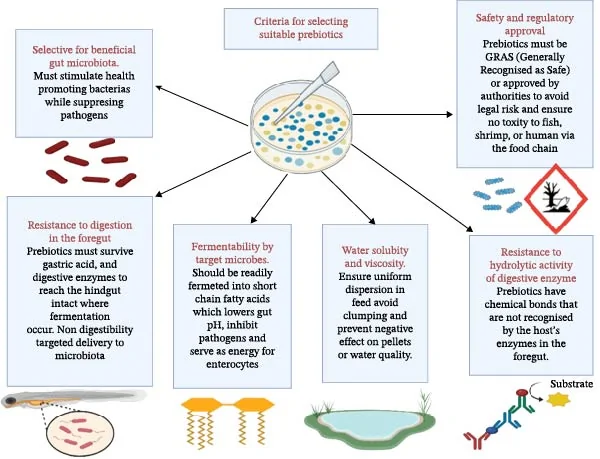
Criteria for selecting suitable prebiotics.

To recognize and authenticate a compound as a prospective prebiotic, it is essential to elucidate its source, origin, purity, chemical composition, and structural traits. Prebiotics must adhere to all relevant national safety criteria and achieve a classification of Generally Recognized As Safe (GRAS) to be approved. They should also undergo thorough assessments for appropriate dosage and potential side effects, be free from contaminants and impurities, and not adversely affect the host’s intestinal microbiota [26]. It is essential to emphasize that the term “prebiotic” should only be applied when modifications to a specific microbiota result in a positive health impact. Food ingredients can be classified as prebiotics based on several key characteristics [120]. First, prebiotics must be resistant to stomach acidity, GIT absorption, and the hydrolytic action of digestive enzymes. Second, they should reach the large intestine, where they are selectively fermented by beneficial bacteria. This fermentation process can lead to modifications in metabolic pathways and enhanced immune function, ultimately contributing to the host’s overall health. A third critical factor is the ability of prebiotics to promote the growth or activity of gut bacteria that support the host’s well‐being. Finally, the technological properties of prebiotics are vital for their effective production and availability for metabolism by intestinal bacteria. By understanding and adhering to these criteria, we can better harness the potential of prebiotics to improve health outcomes (Figure 5).

### 3.3. Types of Prebiotics

Prebiotics are naturally found in cereals, fruits, and legumes [121]. However, commercial chemical and enzymatic processes produce numerous comparable compounds. Inulin, isomaltooligosaccharides (IMO), lactylol, lactosucrose, lactulose, mannan‐oligosaccharides (MOS), oligofructose, transgalactooligosaccharides, arabinoxylan‐oligosaccharides (AXOS), β‐glucans, stachyose, fructo‐oligosaccharides (FOS), galacto‐oligosaccharides (GOS), and xylooligosaccharides (XOS) are among the non‐digestible carbohydrates that prebiotics have been categorized [122]. However, prebiotics differ from carbohydrates, peptides, proteins, and lipids [26]. This list of prebiotic supplements is continually expanding due to the diverse range of species that can be employed to obtain them [123].

A novel class of potential prebiotics is represented by AXOS. These are fragmentation products of arabinoxylans (AX), which are found in the cell walls of many cereal grains. They comprise an O‐2 and/or O‐3‐L‐arabinofuranosyl unit linked to a major chain of beta‐1,4‐linked D‐xylopyranosyl units [124]. The intestinal flora utilizes AXOS as part of dietary fibers, with beneficial effects on various physiological processes. AXOS enhanced the Siberian sturgeon’s immunological responses and growth performance (*Acipenser baerii*) [125]. One of the most widely utilized prebiotics for fish is MOS, which is a glucomannoprotein complex derived from the cell wall of yeast (*Saccharomyces cerevisiae*). Among the several prebiotics, MOS is a significant player, as it has been shown to inhibit the adherence of certain bacteria to the gut and have positive effects on animal growth, intestinal immunity and structure, and gut microbiota [126]. Research indicates that MOS supplementation boosted the body length and weight gain of juvenile tilapia [127]. Natural polysaccharides comprising D‐glucose monomers connected by β‐glycosidic linkages are called β‐glucans. They are structural elements of bacterial, fungal, algal, and plant cell walls and serve as energy storage [128]. The term “glucan” refers to a class of carbohydrate polymers categorized as either α‐ or β‐linked according to their interchain connections. The fundamental structures and conformations of β‐glucans derived from various sources are frequently different. While the conformation of β‐glucans frequently manifests as a random coil, single helix, or triple helix and is influenced by the primary structure, intermolecular force, temperature, and solvent, the primary structure is determined by the type of glycosidic bond as well as the degrees of branching and polymerization.

Several studies have been conducted on the effects of β‐glucan, a well‐known immunomodulator that can be administered to vertebrates by injection, bath therapy, or as food [129]. When given as a prebiotic, β‐glucan increased juvenile red sea bream *Pagrus major*’s immunological responses, digestibility, and feed efficiency [130]. A naturally occurring prebiotic, XOS, is made up of indigestible sugar oligomers based on xylose that are joined by β1‐4 glycosidic linkages. These compounds can be broken down by the lower GIT system into SCFAs, which enhance fish species’ immunological responses and encourage the growth of advantageous *Bifidobacteria* and lactobacilli [131].

### 3.4. Prebiotic Dosage Use

Prebiotics can profoundly impact the bacterial community within the GIT, promote growth rates, and enhance the immune system’s function. Numerous researchers have dedicated their efforts to optimizing the incorporation of prebiotics into animal feed to enhance the growth rates and overall survival. Table 2 presents several key findings from various prebiotic studies, highlighting their significance and potential applications.

**Table 2:** Significance and potential applications of various prebiotics in different fish species.

Prebiotics	Species	Region	Duration	Dosage	Effects	References
MOS	Nile tilapia (*O. niloticus*)	Zagazig University, Egypt	12 weeks	0.5%	Mortality rates of fish under *A. hydrophila* infection challenge were reduced	[49]
Galactomannan oligosaccharides (GMOS)	European sea bass (*Dicentrarchus Labrax*)	University of Las Palmas de Gran Canaria, Spain	63 days	0.5%	Increased the relative abundance of *Bacteroidales*, *Lactobacillales*, and *Clostridiales*	[132]
β‐Glucans	Nile tilapia (*O. niloticus* L., 1758)	Çukurova University, Turkey	2 weeks		Increases leukocyte (WBC) and phagocytic activity levels	[133]
GOS and XOS	Tilapia (*O. niloticus*)	East China Normal University, China	8 weeks	10 g kg^−1^	GOS‐supplementation improved the amino acid composition, while XOS‐supplementation showed beneficial effects on growth performance	[134]
FOS	Asian seabass (*Lates calcarifer*)	Chennai, India	45 days	5 g kg^−1^	Improving the survival rate and immunological parameters	[135]
AXOS	Siberian sturgeon (*Acipenser baerii*)	KU Leuven, Belgium	4 weeks	2%	Improved growth performance and boosted immune responses	[125]
FOS	Stellate sturgeon (*Acipenser stellatus*)	Gorgan province, Iran	75 days	1%	Improved growth performance, beneficial intestinal microbiota, and stimulated immune response	[136]
Inulin	Nile tilapia (*Oreochromis niloticus*)	Abbassa, Egypt	2 months	5 g kg^−1^	Enhanced weight gain, survival, and specific growth rate	[137]

### 3.5. Beneficial Effects of Prebiotics

Prebiotics play a pivotal role in aquaculture, offering numerous benefits for aquatic organisms and the broader agricultural ecosystem. They contribute to the maintenance of a balanced gut microbiota, which is essential for the well‐being of cultured species and a robust immune response [138]. The competition among gut microbes for prebiotics leads to the selective colonization of beneficial bacteria, effectively displacing harmful pathogens from the gut epithelial cells. These beneficial microbes further support gut health by producing SCFAs, which play a crucial role in maintaining gut integrity. Additionally, the modulation of the immune response by gut microbes enhances the functionality of the gut barrier, providing further protection against infections.

### 3.6. Factors Affecting Prebiotic Effectiveness

**Figure 6:**
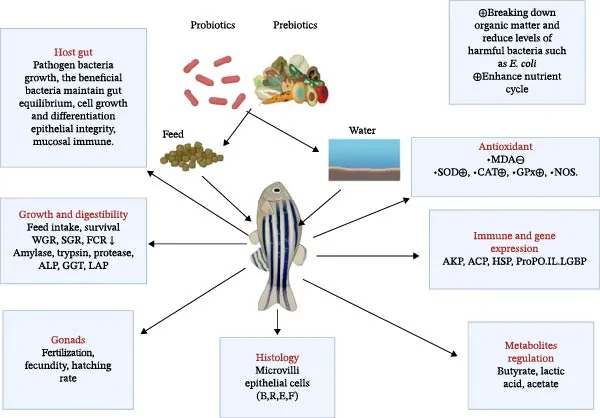
Illustration of the potential benefits of incorporating probiotics and prebiotics in fish farming.

Several factors, including dosage, type, rearing conditions, fish species, and administration methods, influence the effectiveness of prebiotics in fish diets. Dose–response studies play a vital role in identifying dietary prebiotic levels that may be toxic or harmful, as well as those that provide no significant benefits. Moreover, the optimal prebiotic dosage can vary depending on the fish species, size, prebiotic type, and rearing conditions. Therefore, conducting prebiotic dose studies is essential for establishing appropriate feeding regimens that maximize health benefits for each species, age group, and rearing environment. The ability of a fish’s gut microbiota to utilize a prebiotic is contingent upon the specific properties of that prebiotic. For instance, fish species adapted to a plant‐based diet may derive greater benefits from certain types of prebiotics, while carnivorous species may respond better to others. Additionally, prebiotics can enhance the integrity of the gut epithelial barrier, thereby reducing the entry of pathogens and inflammatory agents into the bloodstream. This fortification helps fish avoid systemic infections and lowers their risk of developing inflammatory diseases. The importance of prebiotics and probiotics in fish farming is highlighted in Figure 6.

### 3.7. Additional Factors to Consider in Administering Probiotics and Prebiotics

Numerous probiotics and prebiotics are currently available and being utilized in various industries. While incorporating these products may result in additional costs, the potential benefits include enhanced production efficiency and reduced disease occurrence. An innovative approach involves simultaneously administering substances from both groups, known as symbiotic. The primary objective of a symbiotic is to improve the implantation and survival of live microbial supplements in the GIT [6]. Despite the limited focus on symbiotics in aquaculture, the numerous advantages of probiotics and prebiotics may prompt the development of protocols for delivering these combined substances. This could lead to significant advancements in the industry and potentially revolutionize the way these products are utilized. Additionally, probiotics and prebiotics play a crucial role in promoting a more environmentally friendly and sustainable aquaculture industry by improving water quality and reducing the need for antibiotics.

## 4. Marine Pasture

### 4.1. The Importance of Establishing Marine Pastures

The depletion of marine fishery resources can be attributed to the rapid expansion of the modern fishing industry and its unsustainable practices. This issue is further exacerbated by terrestrial pollution that harms the marine ecological environment. Evidence of this can be seen in phenomena such as increased seawater eutrophication, habitat degradation of marine organisms, and the decline of marine ecosystems [139]. In today’s world, the marine fishery industry is still clinging to antiquated development methods, unable to keep up with the need for top‐notch seafood and a thriving marine ecosystem. This significantly hinders China’s efforts to implement a sustainable development strategy for its marine economy [140]. It is widely recognized that marine aquatic products provide the highest quality of animal proteins. In China, marine aquatic products are a vital food source, offering the population access to high‐quality animal proteins. Establishing “blue granaries” and developing marine pastures presents an innovative approach to fisheries production that aligns with the nation’s goals of restoring marine fishery resources and promoting sustainable economic development. Marine pastures can potentially improve the scientific utilization of marine resources, advance a low‐carbon marine economy, and effectively preserve marine fisheries resources [141].

Marine pastures hold great promise for preserving aquatic biodiversity and improving marine water quality and ecosystems. Additionally, the groundbreaking model for marine fishery production is essential in improving the effectiveness of fishery production and bringing traditional practices into the modern era. The creation of national marine pasture demonstration zones will help the offshore fishing sector secure private investments and expedite the growth of China’s marine pasture industry. This will help propel Chinese marine conservation efforts to advance swiftly and effectively [140].

### 4.2. The Current Status of Marine Pasture Development in China

The development of Chinese marine pastures has a rich history spanning over 30 years. The end of 2019 saw China successfully establish 10 batches of 110 national marine pasture demonstration areas in important regions like the Bohai Sea, Yellow Sea, South China Sea, and East China Sea, based on agricultural data reports, covering a vast area exceeding 200,000 hectares (Table 3). In the near future, there are big goals to finish another 178 national marine pasture demonstration area projects by 2025 [142]. Currently, 11 coastal provinces in China proudly showcase national marine pasture demonstration areas, with Shandong Province taking the lead in investment and construction speed. The impressive Shandong Province now hosts a remarkable 44 national marine pasture demonstration areas, making up 40% of the nation’s total [143]. Artificial reefs, bottom planting, breeding programs, and the subsequent release of marine life are key components in the current construction strategy for marine pasture demonstration areas. However, these methods have a noticeable lack of modernization [142]. The management of marine pasture demonstration areas by marine authorities is somewhat lacking, with oversight often falling under fisheries management. Seafood in the marine pasture primarily consists of shellfish, such as abalone and scallops. Public welfare marine pasture initiatives rely heavily on governmental marine agencies for oversight. Artificial reefs are a key component in these strategies, along with efforts to breed and reintroduce species, with the overarching objective of replenishing marine life and promoting a healthier ocean environment [144].

**Table 3:** Marine ranching construction plan in China.

Constructions	Benefits	Achievements
Requirements for building	Develop the fishing sector by integrating fishery stock improvement, ecological rejuvenation, leisure experiences, tourist attractions, heritage conservation, science education dissemination, and public relations	Creating fresh innovations to support the holistic progression and enduring strength of the oceanic economy, alongside enhancing naval prowess
Yellow Sea and Bohai Sea area construction status at present	With an investment of 4.452 billion yuan, the Yellow Sea and Bohai Sea region established 148 marine ranches spanning 346.7 square kilometers and placed 18.054 million cubic meters of artificial reefs	An intricate system has been developed, incorporating artificial reefs for cultivating sea delicacies, conserving fish populations, nurturing seaweed growth, supporting abalones and sea cucumbers, and promoting recreational fishing
The ongoing construction situation in the East China Sea region	The East China Sea region had committed 383 million yuan to develop marine ranches, establishing 23 across 235.7 square kilometers and introducing 700,000 cubic meters of artificial reefs, expanding the artificial reef zones to cover 206.2 square kilometers	A new marine ranch model has been developed that combines artificial reefs, seaweed beds, and various aquatic species to create a three‐dimensional structure
The present state of construction in Nanhai District	Nanhai District allocated 745 million yuan towards developing marine ranches, creating 74 ranches spanning 270.2 square kilometers, placing 42.191 million cubic meters of artificial reefs, and forming artificial reef zones covering 256.6 square kilometers	Ecological protection

### 4.3. The Construction of Functional Modules

The construction of functional modules is crucial to the contemporary marine pasture technology. This technology encompasses fishery cultivation, ecological conservation, and the development of marine infrastructure, all of which require high technical standards. Modular design is essential for the Marine Pasture project, as it enables the independent construction of various functional modules, including ecological restoration, fishery development, marine environment monitoring, marine tourism, shore, and marine storm warning. These functional modules can then be combined to form the Marine Pasture Project [1]. The use of modular design offers numerous benefits, including reduced marine construction time and reduced risk costs. For example, the competent Department of Marine Pasture can focus on constructing the marine storm warning function module using unified module construction standards. This allows for adding the marine storm warning function module to enterprise construction projects, ensuring that the competent Department of Marine can fulfill their marine storm warning responsibilities without incurring additional monitoring costs. Additionally, enterprises can receive timely marine storm warning information [140]. The marine ranching construction plan in China, including details on its benefits and achievements, is presented in Table 3.

## 5. Methods of Applying Prebiotics and Probiotics in Marine Species

### 5.1. Microencapsulation

Marine species can benefit from the microencapsulation of probiotics and prebiotics to enhance their health and growth performance. In this method, beneficial microorganisms and substrates are encapsulated to protect them from harsh environmental conditions and to improve their delivery and efficacy within the host. The integration of probiotics and prebiotics, often referred to as synbiotics, has shown significant potential to improve the health, immune response, and disease resistance of marine species such as shrimp and fish [145]. Several applications and benefits of microencapsulation in marine aquaculture are discussed in the following sections. *Pacific white shrimp* larvae were fed microencapsulated *Pseudoalteromonas piscicida* (1Ub) combined with MOS, a prebiotic, via enrichment with *Artemia* sp. This method reduced the *Vibrio harveyi* population while enhancing the growth performance, immune responses, and disease resistance. Microencapsulation preserved probiotics from degradation, ensuring their viability during storage and application and thereby optimizing their effects in the shrimp digestive tract. Synbiotic treatment yielded the best results in this study [92]. *Pangasianodon hypophthalmus* was microencapsulated with the probiotic *Bacillus* sp. *NP5* and the prebiotic MOS. Probiotics are protected during digestion and storage by microencapsulation, which makes them more effective [146]. Microencapsulation enhances the application of probiotics and prebiotics in marine species by protecting them during processing, storage, and digestion. By minimizing degradation, this technique ensures that beneficial bacteria, such as *Bacillus subtilis* E20, can effectively modulate the gut microbiota. In marine species such as white shrimp (*Penaeus vannamei*), microencapsulation promotes the proliferation of beneficial bacterial strains while reducing harmful bacteria such as *Vibrio*, thereby improving health, immune response, and metabolic functions [147]. To promote digestive health, microencapsulation can enhance the survival of probiotics and prebiotics, enabling effective colonization of the gut microbiota and modulation. By ensuring adequate delivery of beneficial microorganisms, this method can potentially improve gut health in various species [148].

### 5.2. Bio‐Carrier Inoculation

Bio‐carrier inoculation involves using carriers to deliver beneficial microorganisms to marine organisms, thereby enhancing their resilience and health. This approach has been explored in various marine species, including corals, sponges, and aquaculture‐reared fish and shellfish, to improve stress tolerance and the microbiome composition. Carriers, such as rotifers, alginate beads, and biofilm‐coated substrates, have shown promise in effectively delivering probiotics to target organisms, facilitating microbiome manipulation and environmental remediation. The following sections delve into specific applications and findings related to bio‐carrier inoculation in marine species [149].

#### 5.2.1. Rotifers as Bio‐Carriers for Coral Probiotic Delivery

Rotifers, specifically *Brachionus plicatilis*, have been used as biocarriers for delivering Beneficial Microorganisms for Corals (BMCs) to corals such as *Pocillopora damicornis*. In this method, rotifers consume and accumulate probiotics, after which they were then consumed by corals. This dual‐delivery system offers several ecological implications: it enhances coral resistance to environmental stressors, improves coral health, and provides additional nutritional benefits through the rotifers themselves. Collectively, these effects contribute to the resilience of the overall marine ecosystems [149].

#### 5.2.2. Sponge Extracts as a Bio‐Carrier Strategy for Cyanobacterial Cultivation

In another application, sponge extracts from *Sigmodocia carnosa* have been investigated as a bio‐carrier approach to enhance the growth of sponge‐associated cyanobacterial symbionts. Extracts were incorporated into culture media, resulting in significantly improved biomass production and biochemical constituents in cyanobacteria. This technique not only promotes the cultivation of previously uncultivable marine microorganisms but also reduces sponge waste accumulation along coastal areas, thereby contributing to the health and sustainability of marine ecosystems [150].

#### 5.2.3. Bio‐Carrier Inoculation in Recirculating Aquaculture Systems

Beyond direct delivery to marine organisms, bio‐carrier inoculation has also been applied in recirculatory aquaculture systems (RAS) to improve water quality and system performance [151]. In a study evaluating moving bed biofilm reactors (MBBRs), bio‐carriers were inoculated with mature biofilm (MBI) to accelerate nitrification and stabilize water quality parameters. This approach reduced system start‐up time by 26 days and achieved a stable total ammonia nitrogen (TAN) concentration below 0.5 mg/L within 132 days post‐inoculation. Furthermore, MBI‐treated systems exhibited the highest microbial richness compared to other treatments and the control group, indicating a more robust and resilient biofilter [152]. In summary, bio‐carrier inoculation offers a versatile and effective strategy for delivering beneficial microorganisms to marine species and aquaculture systems. Whether through live carriers such as rotifers, biochemical carriers such as sponge extracts, or engineered carriers such as biofilm‐coated substrates, this approach enhances host health, promotes beneficial microbial interactions, and improves system‐level performance. Future research should focus on optimizing carrier materials, evaluating their long‐term effects on host microbiomes, and scaling up these methods for commercial aquaculture and marine conservation applications.

### 5.3. Artificial Reef Biofilms

Biofilms that develop on artificial biological reef structures, typically composed of concrete and bioactive materials derived from marine algae, are referred to as artificial reef biofilms. These biofilms play a critical role in marine habitat restoration by fulfilling biochemical requirements and supporting the settlement of eukaryotic larvae and algal spores. Compared to natural reefs and control models, artificial reef biofilms exhibit higher microbial diversity and richness, particularly with dominant phyla such as Cyanobacteria, Proteobacteria, and Planctomycetota [153]. Marine biofilms serve as the biological foundation for artificial reefs, transforming submerged structures into a functioning reef ecosystem. Biofilm formation begins when microorganisms adhere to submerged surfaces and release extracellular polymeric substances (EPS), which attract additional microbial colonizers and facilitate community development. However, environmental contaminants such as crude oil and chemical dispersants can disrupt biofilm diversity and function, with cascading effects on higher trophic levels. Understanding the response of these biofilms to environmental disturbances is therefore crucial for mitigating the impacts of oil spills and preserving marine infrastructure [154]. The formation of biofilms on artificial reefs is also essential for the initial recruitment of marine organisms. A study comparing two coral reef sites found that biofilm compositions differed significantly due to environmental factors and anthropogenic impacts. These biofilms, composed of both prokaryotic and eukaryotic organisms, are critically important for coral recruitment and overall reef community health. By creating favorable conditions for reef organism settlement, understanding biofilms can significantly enhance restoration efforts [155]. Submerged structures like historic shipwrecks can also support artificial reef biofilms, providing substrates for microorganisms to attach and form diverse communities. Over time, these biofilms mature through continued EPS deposition, supporting the settlement of both microbes and macrobes and improving corrosion resistance. Nevertheless, environmental disruptions, including oil spills, can alter the biofilm composition and function, potentially affecting the biodiversity and ecological value of these artificial reefs [156]. In summary, artificial reef biofilms are dynamic and ecologically significant communities that underpin the success of artificial reef structures. Their high microbial diversity, recruitment functions, and sensitivity to environmental disturbances make them both valuable tools for habitat restoration and important indicators of ecosystem health.

## 6. Field‐Scale Biotic Applications in Chinese Marine Ranching

Marine ranching was pioneered by China through the establishment of 150 national demonstration zones across various seas by the early 2020s. These zones focus on habitat restoration, stock enhancement, and ecological remediation using biotic organisms such as sea cucumbers, macroalgae, and seagrasses [157]. By integrating artificial habitats into the marine ranching system, ecological carrying capacity is enhanced through the provision of structured environments that support stock enhancement of target species, including the red snail and sea cucumber. Consequently, total yields and stock abundance increase, while negative impacts on ecosystem structure and function were mitigated. Artificial habitats contribute to biodiversity conservation and ecosystem resilience, supporting sustainable marine resource management by facilitating the restoration of depleted fisheries [158].

### 6.1. Sediment Bioremediation in Sea Cucumber Beds (Bohai Bay and Yellow Sea)

#### 6.1.1. Ecological Role of Sea Cucumber in Sediment Bioremediation

Sea cucumbers play a critical ecological role in marine ranching through their deposit feeding activities, which enhance sediment bioremediation and nutrient cycling. By ingesting organic‐rich sediments, sea cucumbers, particularly *Apostichopus japonicus*, promote organic matter mineralization and nitrogen cycling. Through deposit feeding, they increase bacterial abundance and shift microbial communities from producers to decomposers, thereby improving benthic health. Integrated with artificial reefs or integrated multi‐trophic aquaculture (IMTA) systems, sea cucumbers reduce organic buildup and pollution, maintaining a healthier marine ecosystem [159].

#### 6.1.2. Case Study: Bohai Bay

In the Luanhe River estuary, sea cucumber aquaculture farms utilizing stone and artificial reef deployments have created habitats that provide nursery grounds for black rockfish (*Sebastes schlegelii*) since approximately 2013. In addition to enhancing habitat complexity, these farms contribute to sediment turnover and nutrient cycling. In the Bohai Bay region, coastal aquaculture methods, including artificial reef deployment and algal colonization promotion, are used to improve environmental conditions prior to seeding, thereby supporting diverse marine species and strengthening ecosystem resilience [160]. Deposit‐feeding sea cucumbers, such as *Australostichopus mollis*, facilitate bacterial abundance while suppressing microphytobenthos, thereby increasing mineralization and nutrient cycling in coastal sediments. Their bioturbation activities increase the efflux of inorganic nitrogen, which enhances algal productivity while simultaneously reducing the organic matter content. By mitigating the adverse effects of organic and inorganic pollutants, this shift in microbial balance and nutrient dynamics demonstrates the potential role of sea cucumbers in bioremediation efforts, particularly in organically enriched environments such as Bohai Bay [161].

#### 6.1.3. Case Study: Yellow Sea and Shandong Province

In Shandong Province, on the Yellow Sea coast, the Fuhan Marine Ranch has expanded significantly since 2013 through the release of sea cucumbers, shellfish, and algae onto eco‐reefs, which account for ~25% of the ranch’s structures. This system produces tens of thousands of kilograms of sea cucumbers annually while promoting algal growth and bioremediation [162]. Studies have demonstrated that sea cucumber activity in the Yellow Sea enhances benthic biomass and improves sediment conditions through bioturbation and deposit feeding. These activities contribute to sediment turnover, nutrient cycling, and organic matter reduction, all of which enhance benthic habitats and support overall ecosystem health [163, 164].

*Holothuria whitmaei*, a sea cucumber commonly known as the black teatfish, enhances nutrient recycling by bioturbating sediments, which supports benthic microalgal communities and enhances nutrient cycling. Despite only 2% to 14% of sediments being bioturbated by *H*. *whitmaei* each year, their activity significantly increases physical contact with coral reef sediments, which facilitates nutrient release. Sea cucumbers play a crucial ecological role, especially in light of the documented links between their activity and the health of marine ecosystems [165].

#### 6.1.4. IMTA Systems

The Sanggou Bay IMTA system integrates sea cucumbers with kelp and abalone, benefiting the environment through the detritivorous feeding of sea cucumbers on sedimented organic matter. This integration reduces organic waste accumulation, improves water quality, and enhances overall system productivity [162]. In summary, field‐scale biotic applications in Chinese marine ranching, particularly the use of sea cucumbers for sediment bioremediation, have demonstrated significant ecological and economic benefits. Through bioturbation, deposit feeding, and nutrient cycling, sea cucumbers improve benthic health, reduce organic pollution, and support the productivity of coastal ecosystems. The integration of artificial reefs, IMTA systems, and stock enhancement strategies has positioned Chinese marine ranching as a global model for sustainable marine resource management and ecosystem restoration.

### 6.2. Macroalgal Bed (Sargassum/Zostera) and Seagrass Restoration for HAB Mitigation (South China Sea)

#### 6.2.1. Microalgal and Seagrass Bed Ecological Functions, Restoration Techniques, and Artificial Seaweed Reefs (ASRs)

Macroalgal and seagrass beds play a vital role in maintaining water quality and ecosystem health by enhancing water clarity, increasing nutrient uptake, supporting biodiversity, and inhibiting phytoplankton blooms through nutrient competition and allelopathic effects. These habitats also provide critical nursery grounds for marine species, stabilize sediments, and contribute to carbon sequestration (blue carbon) [166]. A variety of techniques are used to integrate microalgal restoration efforts with marine ranching, including planting and transplanting, attaching artificial reefs, and implementing multitrophic systems. ASRs facilitate the establishment of submerged aquatic vegetation beds by fostering ocean macroalgal afforestation, thereby contributing to the overall health and productivity of marine ecosystems. To ensure ASR success, continuous monitoring and refinement are required.

In the South China Sea, pilot projects, approximately nine by the early 2020s, with expansion goals, emphasize the restoration of algae and seagrass beds alongside artificial reefs and stock enhancement. *Sargassum* and *Zostera* (or similar) beds are being restored for multiple ecosystem services, including blue carbon storage, wave attenuation, and harmful algal bloom (HAB) control through nutrient extraction and the provision of habitats for algaecidal bacteria.

Seagrass connectivity studies, particularly those examining dietary and ecosystem links with sea cucumbers, highlight the importance of integrated approaches in marine ranching. For instance, sea ranching of *Stichopus monotuberculatus* has been shown to enhance coral reef ecosystems in Sanya, China. The restoration of sea cucumber beds provides habitat benefits, including reduced eutrophication effects, improved growth performance, and increased survival rates. Additionally, the presence of sea cucumbers may promote seagrass recovery, resulting in clearer waters and a lower risk of HABs [167].

#### 6.2.2. Summary of Field‐Scale Applications and Ecological Significance

Table 1 presents the field‐scale biotic applications and their impacts in Chinese marine ranching from 2018 to 2026.

In summary, field‐scale biotic applications in Chinese marine ranching, particularly the use of sea cucumbers for sediment bioremediation and macroalgal/seagrass restoration for HAB mitigation, have demonstrated significant ecological and economic benefits. Through bioturbation, deposit feeding, nutrient cycling, and habitat provisioning, these biotic approaches improve benthic health, reduce organic pollution, enhance water quality, and support the productivity of coastal ecosystems. The integration of artificial reefs, IMTA systems, and stock enhancement strategies has positioned Chinese marine ranching as a global model for sustainable marine resource management and ecosystem restoration. Table 4 demonstrates the field‐scale biotic applications in Chinese marine ranching from 2018 to 2026 and how they have benefited marine growth in China.

**Table 4:** Field‐scale biotic applications in Chinese marine ranching (2018 to 2026).

Region	Biotic	Key practice (2018 to 2026)	Benefits	References
Bohai Bay	Sea cucumber (*A. japonicus*)	Bottom seeding on artificial reefs; IMTA integration	Sediment processing, nutrient cycling, habitats for finfish, and bioremediation of organics	[160]
Yellow Sea	Sea cucumber + macroalgae	Eco‐reefs, bottom culture (e.g., Fuhan, Sanggou Bay)	Increased biomass (>30% in some cases), organic matter decomposition	[168]
South China Sea	*Sargassum* and *Zostera*, seagrass	Bed restoration, transplantation, and ranching pilots	HAB mitigation via nutrient uptake, competition, biodiversity and carbon benefits: Marine ranching pilot	[169]

## 7. Safeguard Measures of Marine Environment Development in China

**Figure 7:**
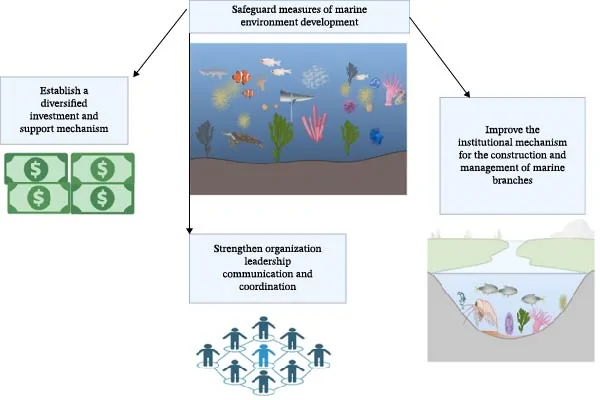
Safeguard measures for marine environmental development.

For almost 60 years, China has had marine protected areas (MPAs). MPAs encompass a wide range of actions specifically designed to preserve biodiversity and offer complete protection for all living within their boundaries, from national parks to fisheries reserves. Because of their ability to protect and restore marine ecosystems, increase their resilience to the effects of climate change, and reap the financial rewards of healthy marine habitats, MPAs are becoming increasingly popular worldwide [170]. Other successful area‐based conservation measures (OECMs) have also recently been recognized by international marine conservation policies as spatial zones that may not fully meet MPA requirements but provide comparative protection for biodiversity and can be used to achieve protected area objectives. Figure 7 summarizes the safeguard measures for marine environment development.

### 7.1. Marine Pasture Network Level

Enhancing the marine pasture network at the network level is crucial for promoting biodiversity and supporting the health of marine ecosystems. By strategically managing and protecting marine pastures, we can ensure the sustainability of marine life and contribute to the overall health of our oceans. Through collaborative efforts and innovative solutions, we can elevate the network level of marine pastures to create a more resilient and thriving marine environment. Collaboration among multiple entities and the incorporation of underwater features are crucial for advancing intelligent marine pastures. Conquering numerous technical obstacles is essential to achieving rigorous benchmarks for ongoing and effective surveillance, widespread spatial reach, accurate and protected input, and evaluative management [171]. By incorporating four‐layer sub‐functions, the information network level strives to create a sensor network for fundamental sensors while integrating suitable network communication protocols and control mechanisms for scheduling [172]. The target is to verify the seamless transfer of essential data for real‐time monitoring, improve the latency performance, and conserve energy. The aggregation layer is crucial in transitioning from centralized cloud computing to edge computing, enabling quick, and accurate data analysis.

### 7.2. Disease Transmission in the Marine Environment

Due to the changing global environment and our growing dependence on marine ecosystems, infectious diseases are increasingly affecting certain species and have the potential to devastate entire populations [173]. These diseases often originate in wild populations, but mariculture poses a higher risk of disease and parasite transmission to wild stocks. This is due to the increased movement and frequency of environmental pathogens and potential reductions in immunity from interbreeding, resulting in billions of dollars in annual costs [174]. Effective disease management is crucial and requires collaboration and efficient information sharing as diseases can spread between wild and farmed populations, imposing financial burdens on both sectors. The likelihood of disease outbreaks is influenced by the number of wild hosts and the intensity of mariculture production [175]. While the transmission of disease from domestic to wild populations is well documented for many species, assessing the impacts at the population level remains challenging, creating a significant information gap. Marine diseases can quickly escalate into emergencies when they have significant ecological, economic, or societal impacts. Enhancing surveillance and developing and continuously refining strategies to mitigate the disease and its consequences will enable us to anticipate and effectively manage these crises [176]. Swift and accurate diagnosis, prediction of disease risks, and real‐time monitoring of environmental factors contributing to the disease spread are crucial for improving surveillance efforts.

The overuse of antibiotics in mariculture, particularly in the fish and shellfish industries, has become a growing concern. Antibiotic residue contamination and widespread antibiotic misuse are significant issues in China. According to research from the WHO, 70% of antibiotics used globally are in Asia, with China responsible for 70% of this consumption [177]. The misuse of antibiotics poses a significant threat to human health and contributes to environmental pollution. Immediate action is necessary to establish policies to regulate and reduce antibiotic misuse and to explore alternative methods to prevent illness in aquatic environments. The use of probiotics and prebiotics to mitigate diseases can be effective if supported by new research and facilitated by communication among researchers, observers, and decision makers through information‐sharing systems. Recent advancements in understanding the risks associated with marine disease may pave the way for the development of policy frameworks that promote effective management and response to marine disease emergencies.

## 8. Enhancing Stress Resilience in Marine Environments Through Probiotics and Prebiotics

**Figure 8:**
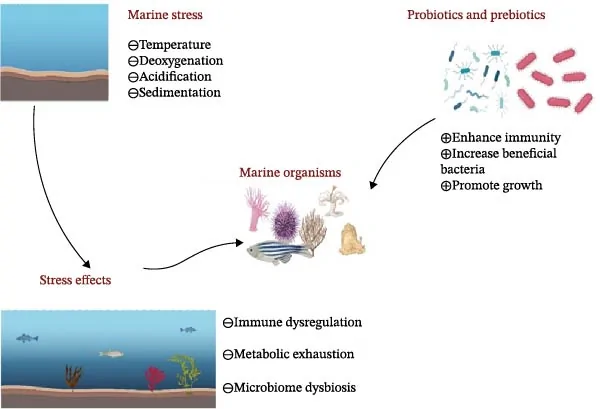
Implementation of probiotics and prebiotics in marine environments to alleviate the harmful consequences of environmental strain.

Various marine animal health issues have been linked to environmental stress. One of the most well‐understood effects of climate change on aquatic diseases is the impact of rising temperatures. Higher temperatures can negatively affect parasites and pathogens, especially if they are exposed to temperatures outside their normal range. This can lead to increased growth of harmful bacteria and heightened pathogenicity, driven by changes in the expression of virulence factors. Temperature changes can also disrupt the microbiomes of marine hosts, potentially leading to immune suppression and increased susceptibility to diseases. Warmer winter sea surface temperatures may allow pathogens to thrive year‐round, potentially causing new diseases or expanding the range of infectious agents. Research has shown that probiotic bacteria could play a crucial role in enhancing the immune system of marine aquatic animals and preventing harmful bacteria from infiltrating the GIT [178]. Probiotic supplementation has been shown to strengthen the immune system and mitigate the negative effects of stress, thereby reducing the likelihood of adverse outcomes (Table 4) [179]. This can be achieved by maintaining a stable digestive system and promoting a healthy gut microbiota through probiotic intervention in water. Numerous studies have highlighted the potential importance of probiotics in the marine environment, particularly in challenging environmental conditions. Probiotics offer a promising solution to combat the detrimental effects of environmental stress on marine animals and enhance their overall health and resilience. Figure 8 illustrates how probiotics and prebiotics can be implemented in marine environments to alleviate the harmful consequences of environmental stress. Table 5 presents research on probiotic supplementation in marine environments to reduce stress levels. Figure 8, on the other hand, illustrates the implementation of probiotics and prebiotics in marine environments to alleviate the harmful consequences of environmental strain (the pressure human activities exert on natural systems, pushing them beyond their regenerative capacity and jeopardizing the ecological balance).

**Table 5:** Probiotic supplementation in marine environments to mitigate stress.

Species name	Probiotics	Stress type	Mitigation	References
Coral *Pocillopora damicornis*	Beneficial microorganisms for corals (BMCs or coral probiotics)	Thermal stress (30°C)	The effects of the pathogen *Vibrio coralliilyticus* can even improve calcification at high temperatures at the skeletal levels	[180]
*Pocillopora damicornis*	Putatively beneficial microorganisms for corals (pBMCs)	Thermal stress (26 and 30°C)	*Vibrio coralliilyticus*	[179]
*Litopenaeus vannamei*	*Bacillus subtilis*		Opportunistic pathogens (two *strains of Vibrio cholerae*, *V. owensii*, two *strains of V*. *rotiferianus*, *V*. *fluvialis*, *Photobacterium damselae subsp. damselae*, *one strain of V. campbellii*, *V. vulnificus*, *and V. parahaemolyticus*)	[181]
Olive flounders (*Paralichthys olivaceus*)	*Lactococcus lactis* WFLU12		Alleviate stress levels in fish	[182]
Hybrid grouper (*Epinephelus fuscoguttatus ♀* × *Epinephelus lanceolatus ♂*)	*Bacillus subtilis* strains		*Vibrio harveyi* stress resilience	[183]
*Litopenaeus vannamei*	*Bacillus* sp. and *Lactobacillus* sp		*Vibrio harveyi* bacterial stress resistance	[184]

## 9. Probiotics and Prebiotics in Marine Ecology

**Figure 9:**
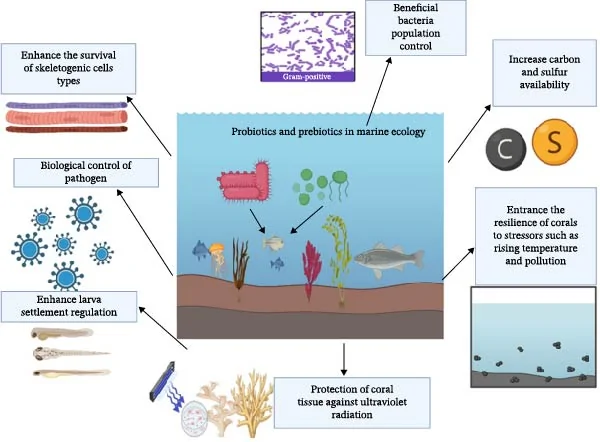
The benefits of probiotics and prebiotics in the marine ecosystem.

Coral reefs play a vital role in marine ecosystems, supporting diverse organisms and providing essential services to society [185]. However, despite their ecological and economic significance, it is projected that by 2030, around 60% of coral reefs worldwide will be at risk due to both global factors, such as ocean warming from increased CO_2_ emissions, as well as local impacts like water pollution, coastal development, and overfishing [186]. While addressing ocean warming and local stressors is crucial for preserving coral reefs, there is a growing emphasis on proactive strategies to bolster the natural resilience of corals to withstand environmental pressures. One such strategy involves manipulating the microbiomes associated with corals, known as BMCs [179]. Though still in its early stages, this approach shows promise in enhancing coral health, potentially boosting resistance and resilience in coral populations, and aiding in the recovery of damaged reefs [187]. BMCs can improve coral fitness through symbiotic relationships with the host, facilitating nutrient cycling within the holobiont [188], and warding off potential pathogens through biological control mechanisms. The concept of the “coral probiotic hypothesis” suggests that microbial communities in coral mucus and tissue layers serve as a defense against pathogens and help corals adapt to changing environmental conditions. Figure 9 provides an illustration of the benefits of probiotics and prebiotics in marine ecosystems.

## 10. A Paradigm Shift: Applying Probiotic/Prebiotic Strategies to China’s Marine Pasture Restoration

The use of probiotics and prebiotics in China’s marine pastures signifies a paradigm shift toward ecosystem‐scale restoration and sustainability, even though they have been widely adopted in conventional aquaculture to improve individual animal health, such as improving the gut microbiota for disease resistance and feed efficiency in confined systems like ponds or cages [189]. In high‐density monoculture settings, conventional aquaculture usually uses these agents through targeted feed supplementation to improve growth performance and decrease antibiotic use, with benefits that are frequently exclusive to the farmed species (e.g., improved survival rates in shrimp or finfish) [190].

Our framework, on the other hand, uses prebiotics (like mannans or inulin) and probiotics (like *Bacillus* and *Lactobacillus* species) not only for host‐level modification but also for more extensive ecological engineering in marine pastures [191]. These open‐water, multispecies habitats, distinguished by artificial reefs, seaweed beds, and fish aggregation devices, support diffuse delivery mechanisms such as substrate‐bound colonization and water column dispersal that support the recruitment of wild stocks and the enhancement of biodiversity beyond farmed populations [192]. This differs fundamentally from standard applications by integrating microbial interventions with national restoration goals, such as those outlined in China’s “Blue Bay” and marine ranching policies, where probiotics/prebiotics facilitate carbon sequestration via enhanced algal‐microbe symbioses and reduce eutrophication through pathogen‐competitive exclusion at the habitat level [193].

## 11. Future Perspective of Marine Pastures

With the emergence of the Smart Earth ideology and the rapid progression of science and technology, there has been a transformative impact on the evolution of marine pastures, moving towards reliance on information and digital intelligence [172]. The smart advancement of contemporary sea fields is an inexorable movement that tackles the deficiencies of conventional human activities in terms of consistency, area coverage, effectiveness, and security. Its involvement is essential in revamping the national fishery sector and fostering sustainable expansion within the marine economy, all while supporting the establishment of a green, low‐carbon ecological system [139]. Integrating traditional acoustic monitoring with localized optics has significantly improved the development of marine pastures, particularly for fisheries cultivation. The underwater acoustic detection method offers high efficiency, speed, minimal environmental impact, and a wide detection range [194]. Various frequency transducers analyze fish characteristics based on specific research parameters, such as size and activity area [195]. Artificial neural networks can train models to identify individual fish using optical images based on their unique features. This technology has enabled the creation of a fish culture tracking system that is effective even in challenging conditions [196]. There is now a system for spotting, following, and tallying fish in slow‐paced underwater settings through low‐resolution video footage. While this model can adaptively update the background, it has a notable false‐detection rate. In the marine environment, wireless technology and sensor networks are essential for improving the efficiency of real‐time monitoring applications [197]. China has started creating marine pastures, but certain constraints must be resolved. Shandong Ocean Pasture incorporates cutting‐edge CAN bus technology [198], enabling real‐time monitoring of multifactorial hydrological data and high‐definition video from the seabed. Despite this progress, obstacles remain, including steep operational and expansion costs, a scarcity of seabed measuring stations, and limitations on depth exploration [199].

## 12. Conclusion

Pathogens are widespread in marine environments, and several outbreaks have caused detrimental effects in aquaculture. Some pathogens not only harm fish species but also the ecosystems, reducing marine productivity and affecting cultural systems. Understanding the implications of these pathogens, accurately diagnosing them, and developing innovative strategies for prevention and control are crucial. China’s traditional marine pasture management pattern is already unable to meet the demands of modern marine pasture health. Probiotics and prebiotics must be implemented as soon as possible to improve modern ocean pasture management conditions. Moreover, multi‐sectoral cooperation must be strengthened, and a marine pasture operation and management mode must be established. Addressing disease is critical to establishing a more robust marine ecosystem, boosting the fish population in the ocean, and ensuring the long‐term success of marine and freshwater fisheries. Improving efforts to prevent and manage aquatic animal diseases is possible by incorporating essential policies into a comprehensive strategy.

The integration of probiotics and prebiotics into China’s marine pasture systems marks a paradigm shift from restorative engineering to microbiome‐driven aquaculture, enabling resilient, antibiotic‐free production at the ecosystem scale. By enhancing juvenile survival, suppressing pathogens, and accelerating bioremediation, these microbial tools are transforming marine ranches from static infrastructure into dynamic, self‐regulating biological platforms that are critical to achieving the Blue Granary vision by 2035. Future perspectives hinge on three interconnected frontiers:

1. Technological Integration: Real‐time eDNA‐based microbiome monitoring, AI‐optimized probiotic dosing, and drone‐enabled reef seeding will enable precision microbial management across vast offshore pastures. Autonomous platforms (e.g., wind‐farm‐integrated bioreactors) could synthesize and deploy host‐specific SynComs (synthetic microbial communities) tailored to local stressors, reducing deployment costs by 60% within a decade.
2. Policy Gaps: Current regulations lack marine‐specific probiotic standards, no mandatory genomic safety screening, efficacy thresholds, or environmental impact assessments for open‐water use. Establishing a National Marine Microbiome Framework under the Ministry of Agriculture and Rural Affairs, aligned with the 14th 5‐Year Plan, is essential to prevent mislabeling, ensure strain traceability, and incentivize adoption through carbon‐credit‐linked subsidies.
3. Research Needs: Priority must be given to field‐scale, multi‐year trials in operational ranches (e.g., Rongcheng and Dalian) to validate long‐term impacts on biodiversity, gene flow, and trophic cascades. Developing native, multi‐functional consortia (e.g., *Bacillus* + *Phaeobacter* + nitrogen‐fixing cyanobacteria) and exploring paraprobiotics and postbiotics for reef applications will close efficacy gaps in high‐salinity, low‐retention environments.

China stands at the threshold of exporting a global microbial blueprint for ocean restoration. With targeted investment in strain banks, digital twin‐ranching models, and public–private microbial alliances, probiotics and prebiotics will not only sustain aquaculture but also engineer the aquatic ecosystem.

## Author Contributions

**Lishuko Ng’onga:** conceptualization, data curation, methodology, formal analysis, writing – original draft. **Kwaku Amoah**: conceptualization, data curation, supervision, methodology, writing ‐ original draft, funding acquisition, writing ‐ review & editing. **Xiaopiao Zhong**, **Yong Zhong**, **Vicent Michael Shija**, and **Peter Mrope:** visualization, writing ‐ review & editing. **Yu Huang**, **Bei Wang**, and **Xiao Jin:** supervision, validation, writing ‐ review & editing. **Jia Cai:** conceptualization, funding acquisition, supervision, methodology.

## Funding

This work is supported by the Guangdong S&T Program (Grant 2025B0202080001), the special project of Guangdong Province for the transformation of science and technology to promote the regional coordinated development of urban‐rural (Grant 2025B0202010041), the Fund of Southern Marine Science and Engineering Guangdong Laboratory (Zhanjiang) (Grant ZJW‐2024‐14), the Science and Technology Plan of Guangdong Province (Grant 2023B0202010016), the Youth Science and Technology Innovation Talent of Guangdong TeZhi Plan Talent (Grant 2023TQ07A888), the Science and Technology Plan of Zhanjiang City (Grant 2024E03007), the National Foreign Expert Program (Category Y) (Grant 110000251420258014), and the Program for Scientific Research Start‐up Funds of Guangdong Ocean University (Grant 060302022310).

## Conflicts of Interest

The authors declare no conflicts of interest.

## Data Availability

No data was generated during the writing of this manuscript.
